# Recommending Breast Cancer Screening to My Mum: Examining the Interplay of Threat, Efficacy, and Virality on Recommendation Intention in the Chinese Context

**DOI:** 10.3390/ijerph20020907

**Published:** 2023-01-04

**Authors:** Chen Luo, Zizhong Zhang, Jing Jin

**Affiliations:** 1 School of Journalism and Communication, Wuhan University, Wuhan 430072, China; 2 School of Journalism and Communication, Tsinghua University, Beijing 100084, China

**Keywords:** eHealth, breast cancer screening, extended parallel process model (EPPM), virality metrics, recommendation intention, message involvement, Chinese women

## Abstract

The burgeoning eHealth campaigns and the emerging daughter-to-mother health communication necessitate a close examination of the intricate mechanism behind recommending preventive behaviors in online settings. The present study addresses existing gaps by investigating how message characteristics and platform-generated virality cues jointly influence younger females’ intention to recommend breast cancer screening to their mothers. Drawing on the extended parallel process model (EPPM) as the theoretical basis, a 2 (threat: low vs. high) × 2 (efficacy: low vs. high) × 2 (virality: low vs. high) randomized between-subjects experiment (*n* = 269) was performed. Results revealed a three-way interaction effect between threat, efficacy, and virality on message involvement. Message involvement was positively associated with recommendation intention and mediated the three-way interaction effect on recommendation intention. This study demonstrates that a high threat can initiate message involvement but fail to trigger recommendation intention. In contrast, a low-threat, high-efficacy, high-virality combination would yield a salutary outcome. Besides, the indispensable role of message involvement in the underlying psychological mechanism behind recommending preventive behaviors was reaffirmed. Theoretical and practical implications are further discussed.

## 1. Introduction

### 1.1. Background

Breast cancer poses grave threats to women worldwide. In China, the speed of the growth in the breast cancer incidence rate is higher than the global average and in Western countries [1]. Early diagnosis has been confirmed as an effective approach to improving the survival rate for breast cancer patients [2]. Among the diagnostic techniques, mammography and ultrasonic breast scanning received wide recognition for their high efficiency and accuracy in detecting potential lesions [3], especially compared to other methods such as breast self-examination (BSE) [4].

Mother–daughter communication is essential in safeguarding women’s health [5]. Previous studies have extensively discussed how mothers promote, educate, or intervene in their daughters’ behaviors regarding certain health issues [6,7]. However, with the rapid growth of social media technologies, the younger generation, which has been lauded as the “digital native generation” [8], is always the recipient of first-hand health messages [9,10]. The intergenerational differences in media consumption patterns challenge the traditional top-down mother-to-daughter communication, and a bottom-up daughter-to-mother communication process emerges in health-related contexts [11]. Although it has become increasingly prevalent for daughters to recommend screening technologies to their mothers [12,13], the driving mechanisms behind the recommendation remain largely under-studied. However, understanding the underlying mechanisms is of great importance because public health practitioners may find a way to count on the younger generation to achieve critical female health objectives and promote advanced medical techniques.

The extended parallel process model (EPPM), as one of the predominant theories in health message design and health behavior change [14,15], may serve as a promising explanatory framework for recommending breast cancer screening to the target population. By presenting the threat and possible solutions to avert the threat, the EPPM guides public health pundits to adopt tailored messages to evoke and alleviate fear by impelling the target group to practice the recommended preventive behaviors [16]. However, the burgeoning eHealth campaign complicates the persuasive process. People are not only exposed to the message content but also to the cues inherent to the platforms [17]. Additionally, the proliferating public health campaigns online (i.e., the eHealth campaigns) require further inquiry about how health messages might interact with online cues to affect messages’ persuasive power. One of the typical online cues is the virality metrics indicating the viral reach of messages [18,19]. Although prevalent, as well as practically meaningful, limited scholarly attention has been paid to how message characteristics and virality metrics jointly influence the persuasive effect of fear-arousing messages. This study aims to address the gap by incorporating EPPM constructs and virality to examine their roles in impacting on female college students’ intention to recommend breast cancer screening (especially mammography and ultrasonic breast scanning) to their mothers in the eHealth setting. We believe this work will inform eHealth promotion strategies and enrich the EPPM theory. Furthermore, message involvement is incorporated as a cognitive mediator to display the comprehensive psychological process behind the breast-cancer screening recommendation.

### 1.2. The EPPM as a Theoretical Basis

EPPM posits that people would initiate threat appraisal and efficacy appraisal after encountering fear-inducing messages, which may lead to different outcomes [20]. Specifically, people will first evaluate the seriousness of the threat (i.e., perceived severity) and whether they are vulnerable (i.e., perceived susceptibility) after exposure to fear appeal messages [21]. If people perceive themselves as insusceptible to the threat, they lack the motivation to proceed and will not respond to the message [22]. On the other hand, if the perceived threat exceeds a certain threshold, fear will be elicited, and people will be motivated to evaluate the effectiveness of the recommended response in averting the threat (i.e., perceived response efficacy) and their ability to perform the response (i.e., perceived self-efficacy) [21,22]. Usually, if the perceived efficacy transcends the perceived threat, individuals will likely engage in the danger control process; otherwise, individuals will reduce their fear by engaging in the fear control process [14].

EPPM has been adopted to explicate breast cancer screening. For instance, Chen and Yang [23] found that Chinese women’s intention to perform BSE was positively associated with threat and efficacy; those who encountered messages featuring high threat and high efficacy simultaneously reported the highest BSE intention. In addition, Termeh Zonouzy and associates [24] proved that Iranian women who read the EPPM-based pamphlets experienced significant improvements in the intention to receive breast cancer examinations. Although EPPM and its constructs have been widely employed in interpreting diverse preventive behaviors, scant scholarly attention has been cast on how EPPM constructs will interact with platform cues to jointly exert persuasive power. However, platform cues are critical for their prevalence in the cyber sphere [25] and their unneglectable role in influencing message processing [26]. Moreover, since public health campaigns increasingly depend on online platforms to facilitate large-scale health promotion [27], and eHealth campaigns can achieve similar behavioral outcomes as health campaigns implemented on traditional media [23], it is imperative for health communication scholars to investigate how message characteristics would interact with platform cues in the eHealth era.

The preceding studies shed valuable light on the interaction between EPPM constructs and platform cues. For example, Kuang and Cho [28] disclosed that the interactivity cue (e.g., hyperlinks on a webpage) strengthens the message involvement in the high-threat, high-response efficacy condition, which results in a higher level of information-seeking intention. Lee-Won and associates [17], on the other hand, indicated that when coupled with high-virality metrics online, the loss-framed health message featuring severity would stimulate greater fear, which in turn leads to colorectal cancer screening intention. Informed by previous efforts, this study takes message involvement—a repeatedly emphasized construct in information processing [29,30] as well as a typical cognitive mediator [31]—into account to check whether EPPM constructs would interact with platform cues to impact message involvement and the distal behavioral intention. Message involvement (which will be detailed in the next section) is an integral part of cognitive responses, and cognitive responses have been proven indispensable in the relationship between health information exposure and health behavior change [32,33]. Hence, our endeavor to build bridges among EPPM, platform cues, message involvement, and the recommendation intention helps to clarify the boundary of EPPM’s effect in the eHealth context and zoom into the psychological process after exposure to EPPM messages on the Internet.

### 1.3. EPPM Constructs and Message Involvement

Conceptualized as the depth of an individual’s attention, comprehension, and elaborate processing at the time of information exposure [34], message involvement has been deemed the motivating factor of persuasion [35]. Message involvement is closely related to the elaboration likelihood model (ELM) proposed by Petty and Cacioppo [35]. The ELM consists of two distinct routes: the central and peripheral routes. The former denotes the deliberate and intensive processing of issue-relevant arguments, while the latter denotes simple inferences based on intuitive cues such as source attractiveness [36,37]. Message involvement serves as the precursor of the two routes [38]. Generally, an increased message involvement can invoke the central route, lead to enduring and resistant attitudes, and trigger behaviors consistent with the attitudes [39]. Accordingly, a large amount of research discloses that message involvement significantly impacts a message’s overall persuasiveness and effectiveness [34,40].

Thus far, only a handful of studies have elucidated the association between the EPPM constructs and message involvement. Block and Keller [41] suggested that due to the coping uncertainty intrinsic to the low efficacy condition, people were prone to assess the trade-off of nonadherence and adherence, leading to a more intensive cognitive engagement. However, using social media metrics as message engagement indicators, Chen and colleagues [32] found that breast cancer prevention information containing both high levels of threat and efficacy had the largest number of readings and likes, which reflects a deeper level of information involvement. On the contrary, Kuang and Cho’s [28] study nullified the main effects of threat or response efficacy in EPPM messages, along with their interaction effect on message involvement. The inconclusive findings necessitate more empirical evidence regarding how EPPM constructs affect message processing in various contexts.

### 1.4. Virality and Message Involvement

As one of the most universal platform cues, virality is inevitable when browsing health information online, which further exerts persuasive effects in tandem with the content [42]. Virality comprises viral reach, affective evaluation, and message deliberation [18]. Among them, viral reach, which refers to the volume of online sharing and forwarding [18], received broad discussion in the extant literature [17,43,44,45]. Scholars argued that virality metrics associate with perceived norms, nurturing a consciousness that the widely disseminated message implies what has been extensively endorsed and what ought to be performed [46]. That is, virality metrics serve as a kind of heuristic cue. People are supposed to jump on the bandwagon to imitate others’ behaviors and spend limited mental resources pondering the content when exposed to high-virality messages [17,44,47,48].

Despite the theoretical rationales, empirical evidence that directly sheds light on how virality metrics influence message involvement is scarce. Trivedi’s [49] survey on viral marketing revealed that different types of viral advertising, including entertaining, informative, and credible, all promoted message involvement among post-80s Internet users. Another study disclosed that most viral advertising features emotional appeal, which prioritizes the peripheral route for message processing and is accompanied by lower requirements for involvement and elaboration [50]. Hence, we aim to enhance the current understanding of virality by investigating how it would affect message involvement, particularly in the case of female health.

### 1.5. Threat, Efficacy, Virality, and Message Involvement

Relevant studies about potential interaction between threat, efficacy, and virality are deficient, making the three-way interaction effect on message involvement largely unknown. Nevertheless, based on existing studies, several reasonable conjectures can be made. For example, the EPPM contends that high threat motivates people to act, and high efficacy directs appropriate actions [22]. Thus, exposure to a high-threat and high-efficacy message would likely stimulate careful inspection of the message to determine what constitutes the threat and what can be done to curb the threat. Furthermore, when coupled with a high level of virality, which represents a cue of social endorsement or social approval [46,51], those already highly motivated may experience a stronger motivation to analyze the message and follow the behavioral instructions. Contrarily, when exposed to a high-threat, low-efficacy condition, people are likely to feel at risk of severe danger but can do little to reverse the situation. Consequently, they may fall into the fear control process, in which defensive avoidance may be elicited [21]. The virality level may hardly function when people intentionally derogate, ignore, or resist the message.

Rival propositions also exist. For example, Kuang and Cho [28] contended that individuals are already motivated to engage in the danger control process in a high-threat and high-efficacy condition. The virality level may not interfere with their involvement since the situation is quite certain and clear. However, in a high-threat and low-efficacy condition, Block and Keller [41] found that the inner uncertainty of low efficacy enticed individuals to scrutinize the message, implying that the fear control process may not be the only way out. Similarly, Rimal and Real [52] concluded that low efficacy might arouse incredulity, motivating individuals either to scrutinize or seek more information to mitigate internal inconsistency. Instead, individuals may devote more cognitive resources to cope with the impending threat, and the virality cues may boost the whole mental activity because of the driving power of perceived social norms.

In light of the above, we propose that threat and efficacy, as two core constructs in the EPPM, would interact with virality to influence individuals’ message involvement when confronting fear appeal messages concerning breast cancer screening.

**Hypothesis 1 (H1).**  *Threat, efficacy, and virality would interact to influence message involvement in the intergenerational breast cancer recommendation context.*

A research question follows to investigate the detailed mechanism regarding the three-way interaction, which has been indecisive and under-studied in previous literature.

**Research Question 1 (RQ1).**  *What are the detailed patterns of the three-way interaction effect among threat, efficacy, and virality on message involvement?*

### 1.6. The Behavioral Outcome of Message Involvement

Message involvement has been proven to be a critical antecedent of behavioral intention and actual behaviors in the health context [53,54]. For example, one study about mammography promotion among African American women found that participants with higher involvement reported a stronger willingness to undergo mammograms [53]. Similar results have been found in HIV/AIDS prevention [55], COVID-19 vaccination promotion [56], weight loss [57], and other topics. Therefore, due to higher message involvement always yielding better health outcomes, we posit that the message involvement effect on behavioral intention is also established when recommending breast cancer screening to others.

**Hypothesis 2 (H2).**  *Message involvement is positively associated with the intention to recommend breast cancer screening to one’s mother.*

Combining the above hypotheses, we postulate that message involvement mediates the relationship between the recommendation intention and the three-way interaction among threat, efficacy, and virality. Therefore, the last hypothesis is formulated.

**Hypothesis 3 (H3).**  *Message involvement would mediate the effect of the three-way interaction among threat, efficacy, and virality on recommendation intention.*

**Figure 1:**
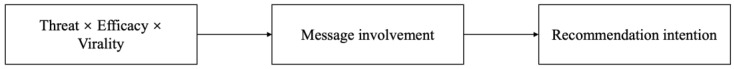
The hypothesized model of this study.

Taken together, the proposed conceptual model is shown in Figure 1.

## 2. Materials and Methods

### 2.1. Design and Participants

This study obtained IRB approval from the corresponding author’s affiliation (Protocol ID: THU202211). An online 2 (threat: low vs. high) by 2 (efficacy: low vs. high) by 2 (virality metrics: low vs. high) between-subjects randomized experiment was performed with participants (i.e., female college students) (*n* = 269) recruited from an online crowdsourcing research platform called *TC Lab* in May 2022. *TC Lab* offers large-scale data collection services and enables researchers to randomly distribute experimental materials and questionnaires to participants [58]. Eligible participants were confined to those who, and whose mothers, had never received any formal medical examinations for breast cancer. Since the most at-risk group for breast cancer in China is women between 45 and 55 years old [59], our participants’ mothers’ ages all lay in the 45–55 range (*M* = 49.64, *SD* = 2.89). For the participants, the average age was 22.62 (*SD* = 2.71). The available demographic characteristics of participants and their mothers are shown in Table 1.

Assuming *p* < 0.05, G*Power showed that the power to detect a medium effect size (*f* = 0.25) in the 2 × 2 × 2 factorial ANCOVA setting was 0.98 when *n* = 269, number of covariates = 5. Thus, it was sufficient to detect medium-size main effects and interaction effects by implementing the current experimental design.

### 2.2. Stimuli

The *Clove Doctor* (*Dingxiang Yisheng* in Chinese), a famous online medical consultation platform in China, was adopted as the template for the message design. Besides regularly updating health-related content, *Clove Doctor* allows users to post questions to seek help from authenticated doctors and search for relevant medical knowledge by entering the disease or drug name. The content on *Clove Doctor* is quite popular, and some posts received substantial sharing in cyberspace [60]. Each participant received a questionnaire link and the webpage screenshot (i.e., an imitation of *Clove Doctor*’s interface) after providing informed consent. Threat was manipulated in the first paragraph following the introductory paragraph, and efficacy was manipulated in the third paragraph. Virality was manipulated by presenting a virality metric at the bottom of the webpage, indicating how many times the article was shared. To ensure information accuracy, all messages were adapted from evidence-based medical information. Each article has the same length of 671 Chinese words to avoid the confounding effect of article length. Participants were debriefed after completion and were told that all information presented was accurate and reliable.

Threat manipulation. In line with previous studies [20,61], the high threat message highlighted breast cancer’s prevalence among women in the age group over 45 (e.g., breast cancer may be the first major cancer type threatening Chinese women aged over 45 in the future) and its devastating effects on women’s health (e.g., stage-four breast cancer comes with great peril with cancer cells metastasizing through the body). In comparison, the low threat message carried less severity and framed breast cancer as a preventable cancer type which is not yet the most prominent cancer type in China. Moreover, we inserted pictures to amplify the contrast between the high- and low-threat conditions [62]. The high threat message was presented with a schematic diagram of mastectomy, while the low threat condition came with an illustration of the pink ribbon (the international symbol of breast cancer awareness).

Efficacy manipulation. Consistent with prior endeavors [20,61], the high efficacy message was composed of high response efficacy and high self-efficacy. Response efficacy stresses the effectiveness of the recommended response in averting the threat (e.g., mammography combined with ultrasound can boost the early breast cancer detection rate). Self-efficacy accentuates one’s ability to perform the recommended response (e.g., Chinese women can easily make an appointment for a breast cancer examination). In the low efficacy condition, we pointed out the weaknesses of breast cancer examination and obstacles in applying for breast cancer examination.

Virality manipulation. Borrowing from former experience [17,44,47], the number of shares was 1003 in the high virality condition and 3 in the low virality condition.

### 2.3. Measures

Perceived threat. The risk behavior diagnosis (RBD) scale, which is theoretically based on the EPPM, was adapted to measure the threat construct [63]. Perceived severity was measured with four items (e.g., “After reading this article, I believe that breast cancer is a severe disease to my mother”). Perceived susceptibility was measured by three statements such as “After reading this article, I think my mother is at risk of getting breast cancer.” All items were measured on a 5-point Likert scale (1 = strongly disagree, 5 = strongly agree) (*M* = 3.69, *SD* = 0.59, Cronbach’s α = 0.79).

Perceived efficacy. Drew on the RBD scale [63] and other studies [23,61], perceived response efficacy was measured with three items (e.g., “After reading this article, I think breast cancer screening works in deterring breast cancer for my mother”). Perceived self-efficacy was measured by four items such as “After reading this article, I think my mother is able to get breast cancer screening to prevent breast cancer”. These items were also measured on a 5-point Likert scale (1 = strongly disagree, 5 = strongly agree) (*M* = 4.11, *SD* = 0.53, Cronbach’s α = 0.81).

Perceived virality. The measurements of perceived virality were modified from Kim’s work [42,44]. Two items from a 5-point Likert scale (1 = strongly disagree; 5 = strongly agree) were employed (e.g., “This article has a large number of shares”) (*M* = 3.23, *SD* = 1.07, Cronbach’s α = 0.80).

Recommendation intention. Following previous experience [64,65], participants were asked how likely they would perform four kinds of behaviors (e.g., “Recommend breast cancer screening to my mother”) on a 5-point Likert scale (1 = very unlikely; 5 = very likely) (*M* = 4.40, *SD* = 0.62, Cronbach’s α = 0.90).

Message involvement. A five item 5-point Likert scale adapted from Cox and Cox [66] was included to gauge message involvement (1 = strongly disagree; 5 = strongly agree). A sample item was “The message made me think” (*M* = 3.88, *SD* = 0.67, Cronbach’s α = 0.84).

Covariates. Due to the concentration of our participants’ ages and their mothers’ ages, there was no need to control for age. Platform use frequency, trust in the platform, and family history of breast cancer were incorporated as covariates [17,47,52,67]. Platform use frequency was asked by one item: “How often do you use online medical consultation platforms (e.g., *Clove Doctor*)?” (1 = Never; 5 = Frequently; *M* = 2.23, *SD* = 0.80). A 7-point semantic differential scale developed by Ohanian [68] was employed to measure trust in the platform (e.g., “The platform is undependable/dependable”; *M* = 4.51, *SD* = 1.07, Cronbach’s α = 0.91). Cancer history was measured with a dichotomous question—“Were any of your family members diagnosed with breast cancer?” A total of 13.0% of the participants had a family member with a breast cancer history. We also asked how a participant was familiar with breast cancer screening [69] and each participant’s annual household income range [70].

## 3. Results

### 3.1. Randomization Check

We performed randomization checks to enable comparable groups and prevent selection bias in treatment assignments. Results showed that the eight groups had no significant differences in the participants’ age (*F*(7, 261) = 1.25, *p* = 0.28), participants’ mother’s age (*F*(7, 261) = 1.17, *p* = 0.32), platform use frequency (*F*(7, 261) = 0.84, *p* = 0.56), family history of breast cancer (*χ*^2^(7)= 5.16, *p* = 0.64), familiarity with breast cancer screening (*F*(7, 261) = 0.93, *p* = 0.48), and annual household income (*F*(7, 261) = 1.71, *p* = 0.11). These statistically non-significant results indicated that our randomization was successful.

### 3.2. Manipulation Check

For the three manipulated factors, independent-samples *t*-tests were conducted for manipulation check. Results indicated that the participants in the high-threat condition perceived a higher level of threat of breast cancer (*M* = 3.81) than those in the low-threat condition (*M* = 3.57), *t*(267) = 3.29, *p* < 0.01, Cohen’s *d* = 0.58. Similar results were also found in efficacy perception (*M*_low_ = 4.04, *M*_high_ = 4.19, *t*(267) = 2.37, *p* < 0.05, Cohen’s *d* = 0.53) and virality perception (*M*_low_ = 2.53, *M*_high_ = 3.84, *t*(267) = 12.73, *p* < 0.001, Cohen’s *d* = 0.84).

### 3.3. Hypotheses Testing

A 2 × 2 × 2 factorial ANCOVA was conducted to test H1. Descriptive statistics for message involvement at different conditions are displayed in Table 2.

**Figure 2:**
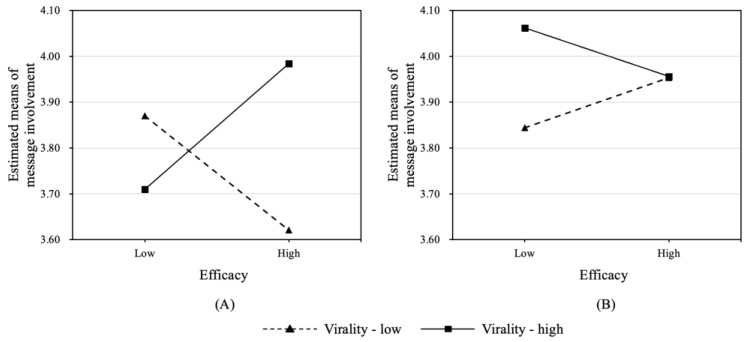
Two-way interaction between efficacy and virality in the (**A**) low-threat; (**B**) high-threat conditions.

Results of ANCOVA indicated a significant three-way interaction effect among threat, efficacy, and virality on message involvement (*F*(1, 256) = 4.87, *p* < 0.05, partial η^2^ = 0.02), lending support to H1. A series of two-way interaction analyses were performed to decompose the three-way interaction effect. We discovered that the interaction between efficacy and virality was significant in the low-threat condition (*F*(1, 124) = 6.33, *p* < 0.05, partial η^2^ = 0.05). In contrast, the interaction was not significant in the high-threat condition (*F*(1, 127) = 0.77, *p* = 0.38, partial η^2^ = 0.01). Figure 2 visualizes the two-way interaction between efficacy and virality in different threat conditions.

Specifically, in the low-threat, low-efficacy condition, the participants did not experience different levels of message involvement when they read messages with high or low virality (*t*(62) = −1.17, *p* = 0.25). However, in the low-threat, high-efficacy condition, the participants who read the high virality message (*M* = 4.01, *SD* = 0.49) were more deeply involved than those reading the low virality one (*M* = 3.66, *SD* = 0.60) (*t*(67) = 2.62, *p* < 0.05). In addition, in the low-threat, high-virality condition, the participants were more involved when reading a high-efficacy message (*M* = 4.01, *SD* = 0.49) than those who read the low-efficacy message (*M* = 3.66, *SD* = 0.73) (*t*(69) = 2.35, *p* < 0.05). However, this did not hold in the low-threat, low-virality condition (*t*(60) = −1.30, *p* = 0.20).

H2 predicts that message involvement would positively affect the recommendation intention about breast cancer screening. A standard multiple linear regression was conducted with all control variables incorporated. The result demonstrated that message involvement positively predicts recommendation intention (*b* = 0.47, *SE* = 0.05, *t* = 9.00, *p* < 0.001), buttressing H2.

H3 postulates that the message involvement would mediate the three-way interaction effect among threat, efficacy, and virality on recommendation intention. Hayes’ [71] PROCESS Macro Model 12 was employed to examine the moderated mediation. Regression results are summarized in Table 3. The 95% confidence interval based on 5000 bootstrap samples revealed that message involvement significantly mediates the three-way interaction effect on recommendation intention (*b* = −0.33, *Boot SE* = 0.15, 95% *CI* = [−0.64, −0.05]). Thus, H3 was supported.

Hayes’ [71] PROCESS Macro Model 3 was further adopted to examine whether message involvement fully mediated the effect of the three-way interaction on recommendation intention. Results demonstrated that there was no significant direct effect of the three-way interaction term on recommendation intention (*b* = 0.01, *SE* = 0.30, *t* = 0.03, *p* = 0.97), supporting the full mediating role of message involvement.

## 4. Discussion

### 4.1. Explanations of the Findings

Under the backdrop of daughter-to-mother health communication and the increasingly prominent eHealth campaigns, this experimental study examines how the EPPM core constructs interact with virality to affect female college students’ intention to recommend breast cancer screening to their mothers. Several findings merit further discussion.

Firstly, the interaction effect of efficacy and virality on message involvement is only significant in the low-threat condition, and the combination of low-threat, high-efficacy, and high-virality can evoke a higher level of message involvement. This finding is somewhat beyond our initial expectations and inconsistent with the fundamental propositions of the EPPM. The multiplicative manner of EPPM advocates that individuals are motivated to take recommended measures to deter threats when they simultaneously perceive high levels of threat and efficacy [72]. Besides, the previous literature also assumes that virality metrics could reinforce the effect of high threat and high efficacy to facilitate a deeper involvement in the fear appeal message [22,46]. A possible explanation for those inconsistencies may be that the participants already know a lot about the seriousness of cancer (e.g., the mean value of perceived severity reached 4.27), just as some studies have suggested before [73,74]. Thus, emphasizing threats in the message may be futile because a high threat level may let people fall into despair and mask the persuasive effects of efficacy and virality. Contrarily, the low threat condition may tone down the already high level of threat perception and exert the potential power of heuristic cues (i.e., virality metrics) to motivate female students to think about screening’s efficacy. This explanation also gains support from the significant main effect of threat. Threat is positively associated with message involvement but fails to predict the ultimate recommendation intention, implying that threat is sufficient to provoke message involvement regarding breast cancer screening. This result conforms to Zhang and Yang’s [75] finding that heightened risk perception of breast cancer can initiate information acquisition as a coping strategy to equip oneself better to tackle the threat. Nevertheless, threat alone is inadequate to activate recommendation due to the absence of recommended responses’ usefulness (i.e., efficacy) and additional persuasive cues (i.e., virality metrics).

Secondly, different from EPPM’s original propositions, which assume that when the threat is perceived as low, individuals have no adequate motivation to further process the fear appeal messages [21]. Our research shows that the “fruitless low threat” is not always the case. Two explanations may account for this. On the one hand, the perceived threat differs from the threat as a message component [76]. The former stands for a cognitive evaluation of the threat contained in the message, while the latter refers to the severity- and susceptibility-related elements presented in the message [76]. Some people may already hold a high threat perception regardless of how much threat is presented in the message. Therefore, the effect of threat manipulation in our study may be confounded by participants’ existing perceived threat of breast cancer. Future studies should differentiate the threat in the message, the perceived threat after viewing the message, the pre-existing perceived threat, and the threat perception change before and after the treatment to estimate the pure effect of experimental manipulation. On the other hand, as the explanations provided previously, low threat may alleviate the psychological stress of breast cancer and works in tandem with high efficacy and high virality. This implies the low threat’s potential to trigger distal outcomes (e.g., recommendation intention) in the severe disease context. Meanwhile, the high threat may be closely related to proximal outcomes (e.g., message involvement). The distinct effects of high threat and low threat merit more scholarly attention because they contribute to different phases in health behavior change.

Thirdly, consistent with the preceding works [22,42], our study reaffirms the significance of efficacy and virality metrics. A higher level of efficacy is necessary to empower at-risk people to overcome the threat [77] and is of paramount importance when individuals already have a high threat perception [22]. Female college students were more involved in the message when high efficacy is presented in the low-threat, high-virality condition, meaning that high efficacy harbors the promise of reducing the health risk. Thus, participants may be driven to chew the message carefully for feasible preventive strategies. Although some previous studies confirmed the information engagement outcome caused by low efficacy [41,52], our study shows a different landscape—high efficacy still matters in the breast cancer issue since it serves as a silver lining in preventing at-risk females from suffering from the serious disease. Meanwhile, the virality metrics are more than aggregated numbers but are psychological hints. The heuristic-systematic model (HSM) offers support for this finding. Chaiken et al. [78] argued that heuristic processing is mentally economic and fits human nature to minimize adopting cognitive resources. Sundar [48] further contended that relying on heuristic processing would be exacerbated when facing overwhelming information online. The virality metrics embedded in the platform represent a collective endorsement, which can be entitled “the bandwagon heuristic” [9,48,79]. People may depend on the bandwagon heuristic and suppose what has been shared frequently is more critical and self-relevant. Therefore, the breast cancer screening message with high virality triggers a perception that breast cancer is a highly-concerned issue and “I” need to evaluate this threat and think about how to combat it.

Fourthly, the three-way interaction effect between message characteristics and platform-generated cues on recommendation intention is only significant indirectly through message involvement, reflecting the unneglectable function of elaboration in inducing recommendation intention. This finding confirms that the cognitive process is indispensable in health persuasion [80]. Mitchell [81] argued that higher message involvement results in intensive attention to and critical analysis of the content. This tendency, to some extent, is similar to the systematic processing defined by Chaiken [82]. It should be noted that the seeming contradiction between the bandwagon heuristic cues and message involvement is untenable in this research. Our results proved that by integrating bandwagon heuristic cues into the EPPM framework, message involvement could be activated for a beneficial health outcome. Therefore, as a manifestation of systematic processing, message involvement is associated with virality cues on the premise that threat and efficacy co-occurred. All the results indicate potential reciprocity between the systematic processing route and the heuristic processing route. Researchers should be keenly aware of this hidden reciprocal relationship and strive to combine cognitive elements and heuristics to pursue an ideal outcome.

### 4.2. Practical Implications

This research sheds light on Internet-based public health campaigns. First of all, to arouse the intention to recommend breast cancer screening, a high-threat message may not be the best option. A viable strategy is to avoid the boomerang effect or defensive avoidance that may be caused by overemphasizing threat [20] and frame breast cancer screening as a well-received, highly efficient medical procedure. Meanwhile, breast cancer should be represented as a preventable threat to strengthen women’s confidence.

Furthermore, virality matters when inducing message involvement in the low-threat, high-efficacy message setting. Prior studies suggested that online messages that are emotionally charged or contain strong visual appeal [83,84] are more likely to go viral. Therefore, public health pundits need to skillfully utilize emotion or visual appeal to enhance persuasive content’s popularity, which helps enhance the probability of in-depth mental processing.

Besides, our results show that threat, efficacy, and virality, along with their interactions, are not directly associated with recommendation intention. Message involvement is indispensable to bridging the association. Hence, public health communicators’ duties are not limited to presenting tailored messages but also stimulating users to think messages over. To fulfill those duties, explicit cues to action need to be valued. For instance, some tips such as “If you find this article helpful, please leave a comment and share it with your friends” could be beneficial to prompt deeper thinking and facilitate health information diffusion.

Additionally, the possibility of a bottom-up daughter-to-mother health communication process is further validated in the current study. Family members, especially offspring, are critical persuaders in breast cancer screening promotion—their involvement and endorsement make recommended health behaviors more credible [85,86]. Therefore, some family-based breast cancer screening seminars or informal family discussions are recommended to improve screening compliance. However, more empirical testimonies are needed to identify what are the most effective strategies to promote daughter–mother communication regarding breast cancer screening.

### 4.3. Limitations and Future Directions

This study is not without defects. First, we only incorporated message involvement, as a cognitive factor, in the mediation process. However, emotional factors, such as fear or anxiety, may also elicit pertinent appraisals that may affect the subsequent recommendation intention [20,87,88]. Researchers should be aware of the power of discrete emotions and build parallel or serial mediation models to test cognitive and affective elements simultaneously. Second, there is, as yet, no consensus on the virality concept. Since the *Clove Doctor* platform only provides content-sharing functions, the current work only considers the viral reach dimension (i.e., the number of shares). Future studies are encouraged to absorb the number of likes (as an indicator of affective evaluation) and the number of comments (as an indicator of message deliberation) as additional virality dimensions [18,19] and replicate this study in various eHealth platforms. Third, the intention–behavior gap is worth further exploring in public health promotion. Thus, whether the recommendation intention of the younger generation can be transformed into the older generation’s actual screening behaviors needs to be investigated in the future. Fourth, we failed to incorporate some important demographic characteristics (e.g., mother’s working background, mother’s health status) and interpersonal communication indicators (e.g., mother–daughter interaction frequency, mother–daughter emotional closeness). These two kinds of information may improve the proposed model’s explanatory power and make the model more compatible with the daughter-to-mother recommendation setting. For instance, emotional dimensions have been stressed in interpersonal health communication and family-based health intervention contexts [89]. A reasonable conjecture would be that a higher level of daughter–mother emotional closeness would trigger a stronger recommendation intention than a lower level. One can better understand how breast cancer screening is recommended in a family by integrating interpersonal communication characteristics with mass communication health message design. Lastly, those experimental stimuli might affect female college students’ intention to receive breast cancer screening. Since they are not among the at-risk population for breast cancer, a long-term follow-up investigation may be warranted to check whether they are more likely to embrace breast cancer screening after entering the high-risk age range.

## 5. Conclusions

By integrating the EPPM and platform cues together, the current study unpacks the interaction between EPPM constructs (i.e., threat, efficacy) and platform cues (i.e., the virality metrics), along with the underlying psychological mechanism behind the interaction and female college students’ intention to recommend breast cancer screening to their mothers. The combination of low threat, high efficacy, and high virality can produce the desired advantageous outcome. Moreover, message involvement fully mediates the relationship between the three-way interaction and recommendation intention, reflecting the importance of cognitive engagement. Our results also revealed that different threat levels are associated with different stages of health behavior change. The findings enhance our understanding of fear appeal messages in the eHealth setting and possess significant implications for women’s health promotion.

## Figures and Tables

**Table 1:** The demographic characteristics of participants and their mothers (*n* = 269).

	Participants	Participants’ Mothers
Demographic characteristics (continuous)	*M* (*SD*)	*M* (*SD*)
Age	22.62 (2.71)	49.64 (2.89)
Annual household income (1 = below 10K CNY; 5 = above 40K CNY)	2.80 (1.45)	2.80 (1.45)
Familiarity with breast cancer (1 = totally uninformed; 5 = well-informed)	2.78 (0.74)	-
Familiarity with breast cancer screening (1 = totally uninformed; 5 = well-informed)	2.32 (0.88)	-
Demographic characteristics (discrete)	*N* (*%*)	*N* (*%*)
Education—Undergraduate level	179 (66.54)	-
Education—Postgraduate level	90 (33.46)	
Education—Primary school or below	-	47 (17.47)
Education—Junior high school	-	99 (36.80)
Education—Senior high school	-	55 (20.45)
Education—College or above	-	68 (25.28)
Family history of breast cancer (0 = False; 1 = True)	35 (13.01)	35 (13.01)

**Table 2:** Descriptive statistics for message involvement at different levels of threat, efficacy, and virality (*n* = 269).

	High Virality		Low Virality		Total	
Threat × efficacy	*n*	*M* (*SD*)	*n*	*M* (*SD*)	*n*	*M* (*SD*)
High threat × high efficacy	36	3.96 (0.94)	32	3.96 (0.64)	68	3.96 (0.81)
High threat × low efficacy	37	4.03 (0.58)	31	3.87 (0.62)	68	3.96 (0.60)
Low threat × high efficacy	39	4.01 (0.49)	30	3.66 (0.60)	69	3.86 (0.57)
Low threat × low efficacy	32	3.66 (0.73)	32	3.86 (0.59)	64	3.76 (0.66)

**Table 3:** Factors predicting message involvement and recommendation intention about breast cancer screening.

	Message Involvement		Recommendation Intention	
Variables	*b* (*SE*)	*t*	*b* (*SE*)	*t*
Constant	3.63 *** (0.20)	18.08	2.55 *** (0.25)	10.11
Platform use frequency	0.02 (0.06)	0.39	0.04 (0.05)	0.78
Trust in the platform	0.07 (0.04)	1.83	−0.01 (0.03)	−0.25
Family history of breast cancer	0.02 (0.12)	0.16	0.08 (0.10)	0.82
Familiarity with breast cancer screening	0.06 (0.05)	1.18	0.02 (0.04)	0.51
Annual household income	−0.09 ** (0.03)	−3.34	−0.03 (0.02)	−1.04
Threat	0.18 * (0.08)	2.25	0.02 (0.07)	0.31
Efficacy	0.03 (0.08)	0.37	0.10 (0.07)	1.55
Virality	0.10 (0.08)	1.23	−0.09 (0.07)	−1.32
Threat × efficacy	−0.04 (0.16)	−0.23	−0.15 (0.14)	−1.14
Threat × virality	−0.00 (0.16)	−0.01	−0.04 (0.13)	−0.31
Efficacy × virality	0.17 (0.16)	1.04	−0.06 (0.13)	−0.47
Threat × efficacy × virality	−0.70 * (0.32)	−2.21	0.34 (0.27)	1.27
Message involvement	-	-	0.47 *** (0.05)	9.00
*R*^2^ (*F*)	0.10 (2.31 **)		0.29 (7.89 **)	

Note. *n* = 269. * *p* < 0.05; ** *p* < 0.01; *** *p* < 0.001.

## Data Availability

The data are available upon reasonable request. For ethical reasons, these data cannot be made public.
